# A Fearful Responsibility

**Published:** 1899-08

**Authors:** 


### EDITORIAL.

### A FEARFUL RESPONSIBILITY.

How many more martyrs from the ranks of the veterinary profession does our Government want placed upon the funeral pile that burns with fearful force upon an ignored profession that has served the country's interest with courage, fidelity, and success?

The death of Dr. Treacy, in Cuba, without rank, recognition, a pension for his family, with not even the privilege or possibilities of a private soldier, is a direful penalty for anyone to make, even as a martyr for the love of his profession, and lays a fearful responsibility on our government, supposedly directed by honest and true men.

For fifteen years faithful at every post of duty assigned him by his superior officers, and bidding the final command of his parental government to die on foreign soil, without as much as the paltry recognition of rank, though a thoroughly professional man. When, we ask, will our government cease to dishonor and degrade a profession that has given more to the enlargement of our commercial supremacy than any other calling, not to speak of what it has done for the preservation and health of our own people, as well as many of those in foreign climes? Higher powers cannot escape their just responsibility for the so-called "Algerism," or, as history will show, rather, "Corbinism," sadly illustrated. Quoting from a letter recently received from one acquainted with the efforts to gain army recognition and their defeat, "As the Army bill passed the House of the last Congress veterinarians were actually commissioned officers, entitled to both retirement and pensions if disabled, but in the Senate the low principles so often seen lately in the War Department were brought into play, and through the adroit wording of the bill by Corbin's immediate military representative in the Senate Committee, the veterinarian or his family, in case of his death, were deprived of any pension, because the actual rank was not given him, thus leaving him a civilian, who is non-pensionable, nor can his family receive even the small pension of a private soldier."

Such, then, is the fate of poor Treacy, and those who must feel his loss most keenly may starve, so far as the parental government of our land is concerned, and a struggling profession that has risen without governmental recognition in our Army Department (faster than any other profession) may continue to sacrifice her chosen sons, as in the past on Western frontiers in Indian raids, to-day in climes that are pest-ridden to even those who are native to those soils. With every intelligent nation of the earth according rank, pay, allowance, with provision for disability and pension in the event of death to those who survive, to their army veterinary service the United States stands out singly and alone in denying these well-deserved and more-than-earned rights, and upon the shoulders of those who have studiously denied the same there rests a fearful responsibility, and on the fair name of our land a blot and a disgrace.

